# Tellurium Compounds Prevent and Reverse Type-1 Diabetes in NOD Mice by Modulating α4β7 Integrin Activity, IL-1β, and T Regulatory Cells

**DOI:** 10.3389/fimmu.2019.00979

**Published:** 2019-05-29

**Authors:** Tom Eitan Yossipof, Ziva Roy Bazak, Dvora Kenigsbuch-Sredni, Rachel R. Caspi, Yona Kalechman, Benjamin Sredni

**Affiliations:** ^1^The Mina & Everard Goodman Faculty of Life Sciences, The Safdiè AIDS and Immunology Research Center, C.A.I.R. Institute, Ramat Gan, Israel; ^2^Interdisciplinary Department, Bar-Ilan University, Ramat Gan, Israel; ^3^Laboratory of Immunology, National Eye Institute, National Institutes of Health, Bethesda, MD, United States

**Keywords:** diabetes, tellurium, integrin, inflammation, IL-1β, Tregs

## Abstract

The study shows that treatment of NOD mice with either of two tellurium-based small molecules, AS101 [ammonium trichloro(dioxoethylene-o,o')tellurate] or SAS [octa-O-bis-(R,R)-tartarate ditellurane] could preserve β cells function and mass. These beneficial effects were reflected in decreased incidence of diabetes, improved glucose clearance, preservation of body weight, and increased survival. The normal glucose levels were associated with increased insulin levels, preservation of β cell mass and increased islet size. Importantly, this protective activity could be demonstrated when the compounds were administered either at the early pre-diabetic phase with no or initial insulitis, at the pre-diabetic stage with advanced insulitis, or even at the advanced, overtly diabetic stage. We further demonstrate that both tellurium compounds prevent migration of autoimmune lymphocytes to the pancreas, via inhibition of the α4β7 integrin activity. Indeed, the decreased migration resulted in diminished pancreatic islets damage both with respect to their size, β cell function, and caspase-3 activity, the hallmark of apoptosis. Most importantly, AS101 and SAS significantly elevated the number of T regulatory cells in the pancreas, thus potentially controlling the autoimmune process. We show that the compounds inhibit pancreatic caspase-1 activity followed by decreased levels of the inflammatory cytokines IL-1β and IL-17 in the pancreas. These properties enable the compounds to increase the proportion of Tregs in the pancreatic lymph nodes. AS101 and SAS have been previously shown to regulate specific integrins through a unique redox mechanism. Our current results suggest that amelioration of disease in NOD mice by this unique mechanism is due to decreased infiltration of pancreatic islets combined with increased immune regulation, leading to decreased inflammation within the islets. As these tellurium compounds show remarkable lack of toxicity in clinical trials (AS101) and pre-clinical studies (SAS), they may be suitable for the treatment of type-1 diabetes.

## Introduction

Type 1 diabetes mellitus (T1D) develops as a result of pancreatic islet β-cells loss, mainly by means of CD4 T cells and other leukocytes action. The autoimmune etiology of T1D is affected by both genetic and environmental factors. Nevertheless, the exact mechanisms of disease initiation remain largely unknown.

There is no proven means to avoid the development of diabetes, nor to control the anti-β-cell autoimmune response after the disease has been diagnosed. Furthermore, the progressive loss of self-tolerance to islet β-cell antigens in human patients and animal models, is one of the most important fundamental contributing factors to T1D development. One of the main primary factors controlling such self-tolerance is the activity of Regulatory T cells (Tregs) (1).

Upon immunological challenge, lymphocytes migrate from the bloodstream into secondary lymphoid tissues such as lymph nodes (LNs) and Peyer's patches. Infiltration of immune cells is essential for organ and antigen-specific adoptive immunity against pathogens and antigens, including autoimmunity.

This migration is controlled in part by adhesion molecules and chemokine receptors on lymphocytes and their ligands on blood vessel high endothelial venules (HEVs) in LNs and Peyer's patches (2–4).

In T1D, activated CD4^+^ and CD8^+^ T lymphocytes destroy the insulin-producing β-cells of the pancreatic islets. In recent years it was recognized that B cells in pancreatic LNs are crucial antigen presenting cells (APCs) not only to initiate and optimize priming of naive autoreactive CD4^+^ T cells by β cell antigens, but also to contribute to epitope spreading of T cell autoimmunity among β cell antigens (5). Furthermore, the α4β7 integrin/MAdCAM-1 pathway has been found crucial for the migration of B cells from the bloodstream into pancreatic lymph nodes (PLN) in NOD mice (6). Importantly, an α4-integrin-VCAM-1 interaction contributes to T cell entry into the pancreatic islets and in the pathogenesis of T1D (7).

AS101 is a potent tellurium-based immunomodulator, which has demonstrated activity for several indications, both *in vitro* and *in vivo*, and has a large number of potential therapeutic applications (8, 9). The compound is non-toxic and is currently undergoing phase II/III testing in patients with cervical tumors, and phase I/II testing for treatment of patients with age-related macular degeneration. Much of the biological activity of AS101 can be attributed to its chemical redox interactions with vicinal thiols in the exofacial domain of VLA-4 (10); these modifications enable this agent to mediate diverse effects including abrogation of acquired drug resistance in acute myelogenous leukemia (10), and improvement of symptoms in experimental autoimmune encephalomyelitis (11) and uveitis (12). The specific redox-modulating activities of AS101 result in a variety of beneficial biological effects, demonstrated in diverse preclinical and clinical studies (13). The anti-inflammatory properties were found to be crucial for the clinical activities of AS101, including the protective effects of AS101 in autoimmune diseases (14–17) and in septic mice (18). The same thiol–redox interactions of AS101 enabled it to exert beneficial effects in a variety of tumor models in mice and humans where AS101 had clear antitumor effects (10, 19, 20).

The second generation tellurium IV compound SAS synthesized by us, has also been shown to possess a unique Te^IV^-thiol chemistry and exert biological activities similar to those of AS101 (13).

Based on our previous data showing that AS101 inhibits the α4β7 activity in mesenteric lymph node cells of DSS-induced murine colitis (14), we hypothesized that both tellurium compounds may have the potential to suppress the activity of the α4β7 integrin on lymphocytes from NOD mice, and consequently prevent their infiltration into pancreatic LNs, potentially affecting the pathogenesis of T1D.

We demonstrate that AS101 and SAS are able to prevent as well as inverse new onset type 1 diabetes in the NOD mouse model, and exhibit effects on α4β7 activity and lymphocyte migration, as well as inhibition of pancreatic IL-1β and IL-17 production and enrichment of T-regulatory (Treg) cells in the islets. Our data thus suggest that tellurium compounds may have potential in the treatment of type 1 diabetes.

## Methods

### Antibodies and Reagents

FITC anti-mouse CD3; PE anti-human/mouse Integrin β7 (Biolegend, San Diego, CA, USA); APC anti-mouse CD45R/B220; anti-Mouse/Rat Foxp3 PE; anti-Mouse CD4 FITC; anti-Mouse CD25 APC; rat IgG2a Isotype Control PE (eBioscience, Carlsbad, CA, USA); Alexa 594 anti-mouse; Alexa 488 anti-rabbit; anti-glucagon; anti-insulin (Innovex Biosciences, Richmond, CA, USA); recombinant mouse MAdCAM-1 Fc chimera (R&D BioSystems, Minneapolis, MN, USA); murine TNF-α (Peprotech, Rocky Hill, NJ, USA); mouse IL-1β, IFNγ, and IL-17 ELISA kits (R&D Biosystems). AS101 and SAS were supplied by M. Albeck, Bar-Ilan University, Ramat-Gan, Israel, and were synthesized at Bar-Ilan University.

### Mice

Three to Four week old NOD/ShiLtJ (strain 001976) female mice were purchased from the Jackson Laboratory (Bar Harbor, ME), bred, and housed in individual metabolic cages with unlimited access to food and water. Experiments conformed to approved institutional protocols and were approved by the Institutional Animal Care and Use Committee.

### Cells

SVEC-4 murine endothelial cells were obtained from the American Type Culture Collection. Cells were cultured in DMEM containing 10% fetal calf serum at 37°C with 5% CO_2_ and 95% air.

### Purification of Tregs and T Effector Cells

CD4^+^CD25^+^ Treg were purified from 3 months old NOD mice using a CD4^+^CD25^+^ Treg purification kit (Miltenyi Biotec) according to the manufacturer's instructions, with slight modifications. The CS columns were used for depleting non-T cells, and LS columns were employed to positively select CD4^+^CD25^+^ cells. Non-CD4 cells were magnetically labeled using a cocktail of biotin-conjugated antibodies recognizing CD8, CD11b, CD45R, CD49b, Ter-119, followed by incubation with anti-biotin micro beads. The labeled cells were then depleted over a column. To attain maximum purity, the CD25 positive selection was performed twice. CD4^+^CD25^−^ T cells were isolated from the CD25-depleted fraction using CD4 microbeads and LS columns.

### Cell Migration Assays

Cells (2.5 × 10^5^) were incubated for 1 h in the presence of mobilized MadCAM-1 (100 μg/ml) medium containing MnCl2 (0.25 mM) and various concentrations of SAS or AS101. The cells were washed twice with PBS, and equal numbers of cells were loaded onto 8 mm polycarbonate membrane inserts. The bottom chambers were filled with RPMI-1640 (800 μl) with FBS (20%), containing MadCAM-1 (100 μg/ml) as a chemoattractant. Cell migration was quantified after 24 h by Trypan Blue exclusion test using a hemacytometer.

### FACS Analysis

For evaluation of integrin expression on lymphocyte suspensions, single cells from spleens, peripheral LNs, and PLNs from 8 week old NOD mice were stained with FITC-conjugated anti CD3, PE-conjugated anti β7, and APC-conjugated anti B220. Cells were then analyzed on a BD FACSCalibur flow cytometer. The proportion of B and T cells that express the integrin was evaluated on 1 × 10^4^ B220 B or CD3 T cells in the lymphocyte forward scatter/side scatter gate. For evaluation of the proportion of Tregs, lymphocytes from PLNs from 20 w old treated mice were isolated and stained for membranal CD4 (FITC) and CD25 (APC) and nuclear Foxp3 (PE). Cells positive for both CD4 and CD25 were gated, and the expression of Foxp3 on this subpopulation was evaluated. The percentage of CD4^+^CD25^+^Foxp3^+^ cells from total lymphocytes was evaluated.

### Quantitation of Caspase Activity

Isolated pancreatic extracts from treated mice were prepared in Tris/acetate buffer (pH 7.5) at 30°C. The extracts were centrifuged at 12,000 × *g* for 10 min, and the supernatant was collected. A volume of supernatant equivalent to 100 μg of protein was assayed for caspase-1 or caspase-3 activity using colorimetric caspase-1 and−3 assay kits (R&D Biosystems).

### Quantitation of Cytokine Levels

IL-1β, IFNγ, and IL-17 ELISA kits were used for the quantitative measurement of these cytokines in pancreas extracts of treated mice.

### Attachment Assay for Evaluation of the α4β7 Activity

For testing attachment to MadCAM-1, 96-well plates were coated with 80 μL of MAdCAM1 (100 μg/ml), or BSA. Cells were incubated in the wells for 2 h in the presence or absence of AS101 or SAS followed by extensive (3x) washing. The proportion of cells that remained attached to the wells was determined by the colorimetric XTT (2,3-bis[2-methoxy-4-nitro-S-sulfophenynl]H-tetrazolium-5-carboxanilide) assay at 450 nm.

For testing attachment to endothelial cells, SVEC-4 endothelial cells were cultured on 6 well plates until 80% confluency. Next, 10 μl of rTNFα (20 ng/ml) was added to the wells to induce MadCAM-1 expression, and then washed. Lymphocytes at 1 × 10^6^/well were then added in the presence or absence of AS101 or SAS and incubated o.n. Wells were washed with PBS. The remaining attached cells were stained with anti CD3 and anti B220. Endothelial cells are not stained by either antibody (Supplementary Figure 1). The proportion of T or B cells in the total cell population was recorded.

### Islet Histology

Resected pancreas heads were fixed in 4% formalin and then paraffinized. To prepare histological sections, 5 μM sections were cut from each paraffin block, and stained with hematoxylin-eosin for detection of insulitis.

The level of insulitis was determined according to the following scale: Grade 0 - no insulitis (no infiltration); Grade 1- pre-insulitis (<25% of the islet area infiltrated); Grade 2- mild insulitis (<50% of the islet area infiltrated); Grade 3 – severe insulitis (>50% of the islet area infiltrated).

### Immunohistochemistry: Insulin and Glucagon Staining

Fixed paraffin embedded pancreas head sections were deparaffinized and rehydrated. After antigen retrieval, slides were incubated with anti-mouse insulin Ab and anti-glucagon Ab o.n. at 4°C, followed by secondary Alexa 594-conjugated and Alexa 488-conjugated secondary antibodies for 1 h at RT. This was followed by Hoechst staining. Ten slides/mouse were visualized by fluorescence microscopy.

### I.P Glucose Tolerance Test

After 12 h fast, mice were administered an i.p bolus of 2 g/kg glucose Blood glucose concentrations were measured by tail bleed at the indicated time points before and after glucose administration. All glucose measurements were performed using a Free Style Freedom (Alameda, CA, USA) glucometer.

### Adoptive Transfer

NOD mice (5 w old) were treated with PBS or with 1 mg/kg AS101 every other day. Mice were mated, and treatment continued during gestation and during lactation, until the offspring reached an age of 3 weeks. At that time treatment stopped. Some of the male offspring of treated mothers were irradiated at 6w of age with 550 rads and were injected 24 h later with 20 × 10^6^ splenocytes from diabetic female NOD mice.

### Statistics

Data are presented as means ± SE. For comparisons between groups in the *in vitro* and in some *in vivo* studies, we used the one- or two-way ANOVA. For *in vivo* studies (weight, blood glucose, glucose tolerance test, insulitis), ANOVA multiple comparisons with repeated measures with Bonferroni corrections, were applied. For analysis of incidence of diabetes and survival, the Kaplan Meier survival analysis tests were applied. The software used for all statistical analysis was IBM SPSS Statistics 21. *p* < 0.05 was considered statistically significant.

## Results

### AS101 and SAS Attenuate α4β7 Activity in Mononuclear Cells Isolated From NOD Mice

**Figure 1:**
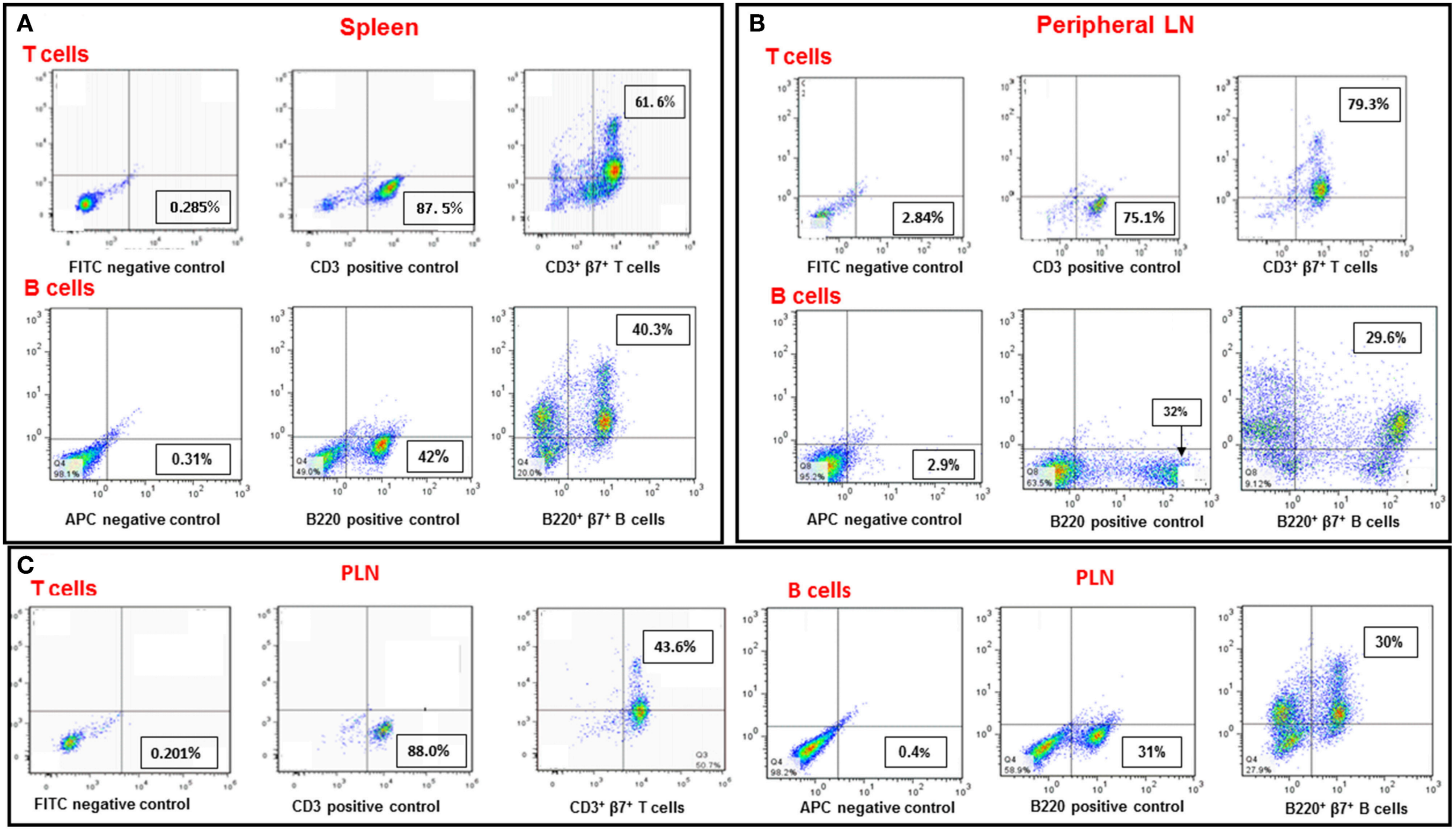
Expression of the β7 integrin subunit on T and B cells in various organs of NOD mice. Single cell suspensions from spleens **(A)**, peripheral LN **(B)**, and PLN **(C)** from 8-week-old NOD mice were stained with FITC-conjugated anti CD3, PE-conjugated anti β7 and APC-conjugated anti B220. CD3^+^β7^+^ positive cells represent T cells expressing the β7 integrin subunit. B220^+^β7^+^ positive cells represent B cells expressing the β7 integrin subunit. The results represent one experiment representative of three performed.

**Figure 2:**
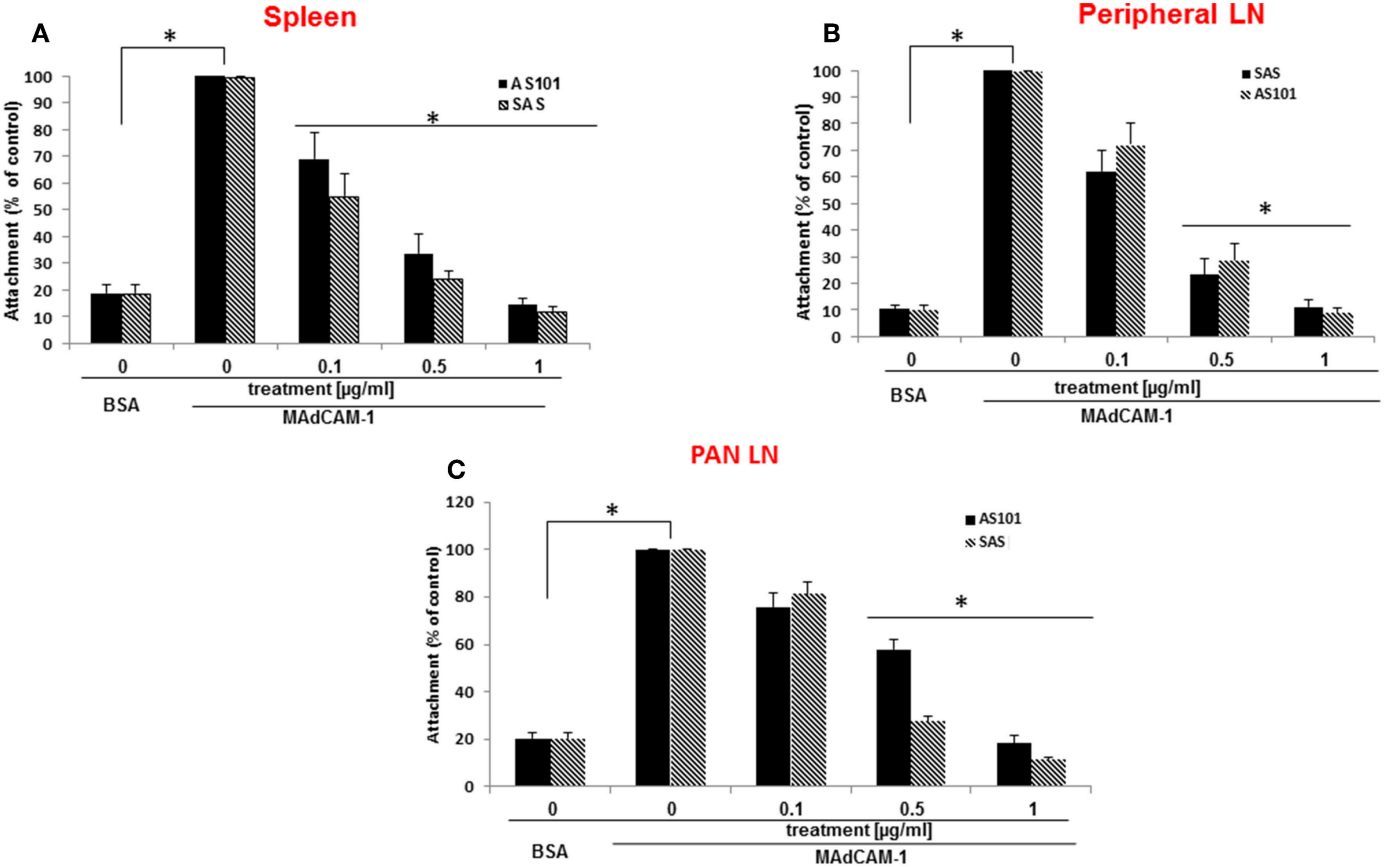
AS101 and SAS inhibit *in vitro* the activity of the α4β7 integrin in various organs of NOD mice. 5 × 10^5^ single cells from spleens **(A)**, peripheral LN **(B)**, and PLN **(C)** from 10 week old NOD mice were cultured on BSA or MAdCAM-1-coated micro wells for 2 h in the presence or absence of different concentrations of SAS or AS101The cells were washed twice and then subjected to XTT reagent according to the manufacturer's instructions. The results represent means±SE of three different experiments. ^*^p < 0.001 decrease vs. MAdCAM-1 without AS101 or SAS (SAS 0).

**Figure 3:**
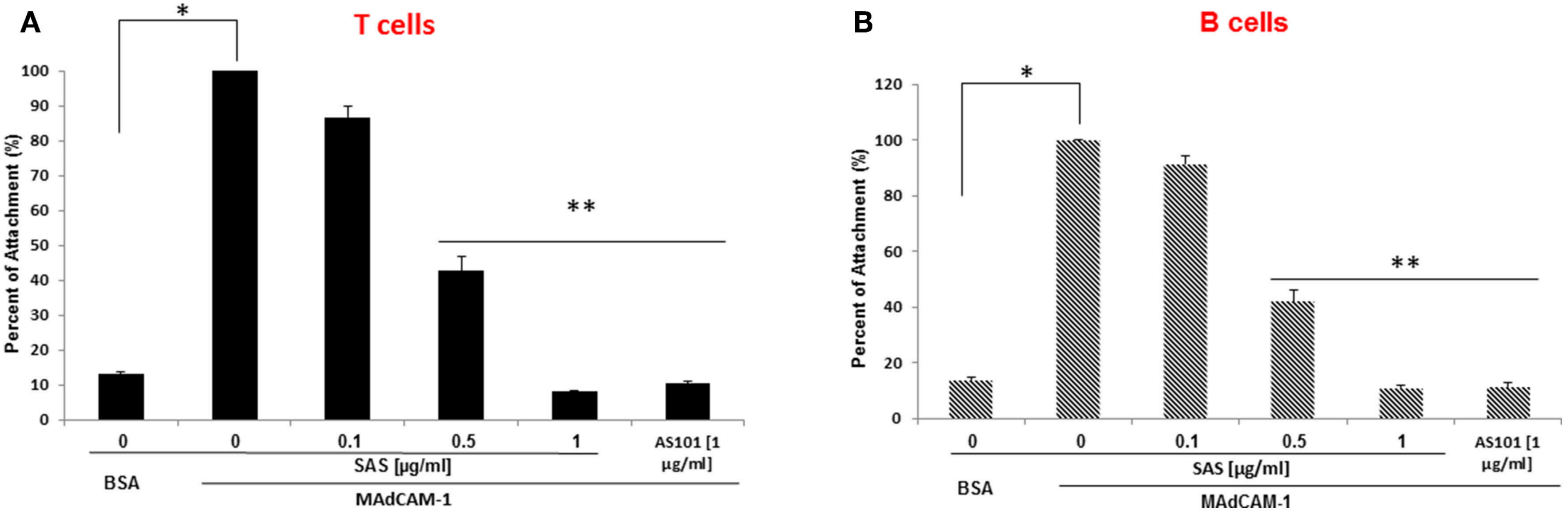
AS101 and SAS inhibit *in vitro* the activity of the a4β7 integrin on both T and B spleen cells of NOD mice. T and B spleen cells from 10 week old mice were sorted by FACS, using anti CD3 or anti B220 antibodies. Single cells (5 × 10^5^) of each lineage (**A** = T cells; **B** = B cells) were cultured on BSA or MAdCAM-1-coated microwells for 2 h in the presence or absence of different concentrations of SAS or 1 μg/ml AS101. The cells were washed twice and then subjected to the XTT reagents according to the manufacturer's instructions. The results represent means ± SE from three different experiments. ^*^p < 0.01; ^**^p < 0.001 decrease vs. MAdCAM-1 without AS101 or SAS (SAS 0).

The α4β7 integrin, MAdCAM-1 pathway is essential for infiltration of lymphocytes into pancreatic islets, and for the initiation of T1D in NOD mice (21).Therefore, regulation of this pathway has the potential to ameliorate the course of the disease. For this purpose, we first analyzed β7 integrin expression on T and B cells from various organs of NOD mice. The results shown in Figure 1 demonstrate that most spleen (Figure 1A) and peripheral lymph node (Figure 1B) T and B cells express the α4β7 integrin, while about 50% of pancreatic T cells (Figure 1C), but most of B cells in this organ still express this integrin. We then analyzed the ability of AS101 and SAS to inhibit the activity of α4β7 on T and B cells from various organs of NOD mice. This was performed by testing the level of attached cells to the α4β7 specific ligand, MADCAM-1. Figure 2 shows a dose dependent and significant inhibition of the α4β7 integrin activity by both compounds. This was evident in both total spleen cells (Figure 2A), peripheral lymph node cells (Figure 2B), and pancreatic lymph node cells (Figure 2C). Moreover, SAS significantly inhibited the activity of the integrin in sorted T (Figure 3A), and B (Figure 3B) splenocytes (Figure 3).

**Figure 4:**
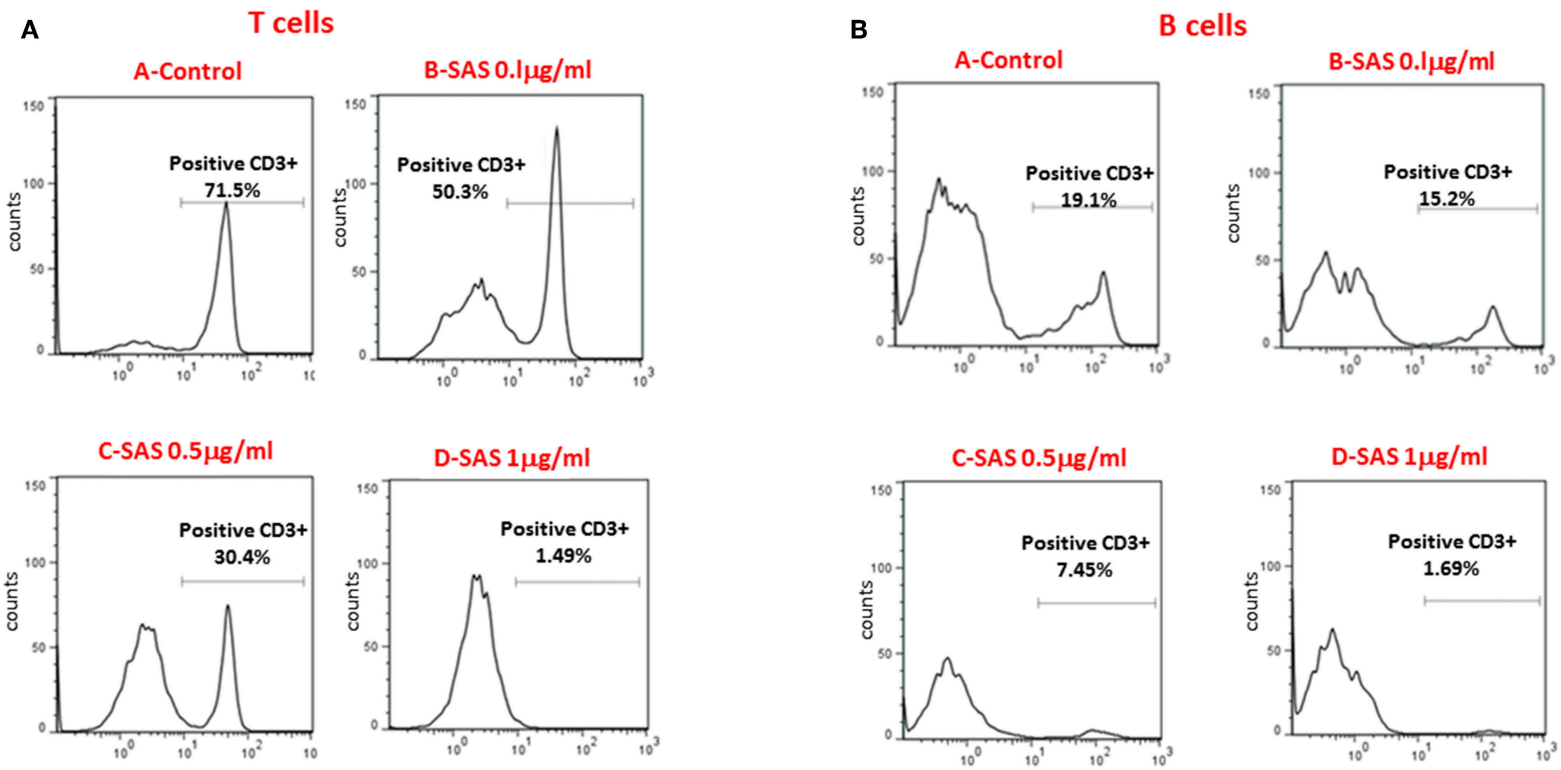
SAS inhibits *in vitro* attachment of T and B lymphocytes from NOD mice to the high endothelial venular ('HEV') cells (SVEC-4)**.** Cells were washed twice. Eighty percent confluent endothelial SVEC cells in 6 well plates were stimulated with TNFα to induce MAdCAM-1 expression. After 10 h, 3 × 10^6^ splenocytes from 10 week old NOD mice were added in the presence or absence of various concentrations of SAS o.n. Culture plates were washed and the attached cells were removed and stained with FITC-conjugated anti CD3, and APC-conjugated anti B220 antibodies for the detection of attached T **(A)** or B **(B)** cells. The results represent one experiment, representative of three performed.

To further substantiate the inhibitory effect of SAS on integrins, we examined the extent of lymphocyte adhesion, to high endothelial venular ('HEV') cells. MAdCAM-1 is expressed on approximately 95% of HEVs on the lymph nodes of young NOD mice (6). The interaction between MAdCAM-1 on HEVs in PLN and α4β7 on lymphocytes enables their trafficking to PLN. For these experiments, we used the SVEC-4 endothelial cell line which represents HEV cells and highly expresses MADCAM-1 after exposure to TNFα (22). Figure 4 shows that treatment with SAS significantly inhibits attachment of both T and B cells from NOD mice to endothelial cells, and that the effect is dose–dependent. Collectively, these results suggest that by inhibiting the interaction between α4β7 on T or B lymphocytes from NOD mice, and MADCAM-1 on endothelial cells, AS101 and SAS might affect attachment and penetration of autoreactive T and B cells through pancreatic high endothelial venules, potentially affecting the course of T1D. For this purpose, we used the NOD mouse model of T1D using AS101 and SAS in various concentrations and different timing of administration.

### Treatment of NOD Mice With AS101 Alleviates Clinical Symptoms of Diabetes

**Figure 5:**
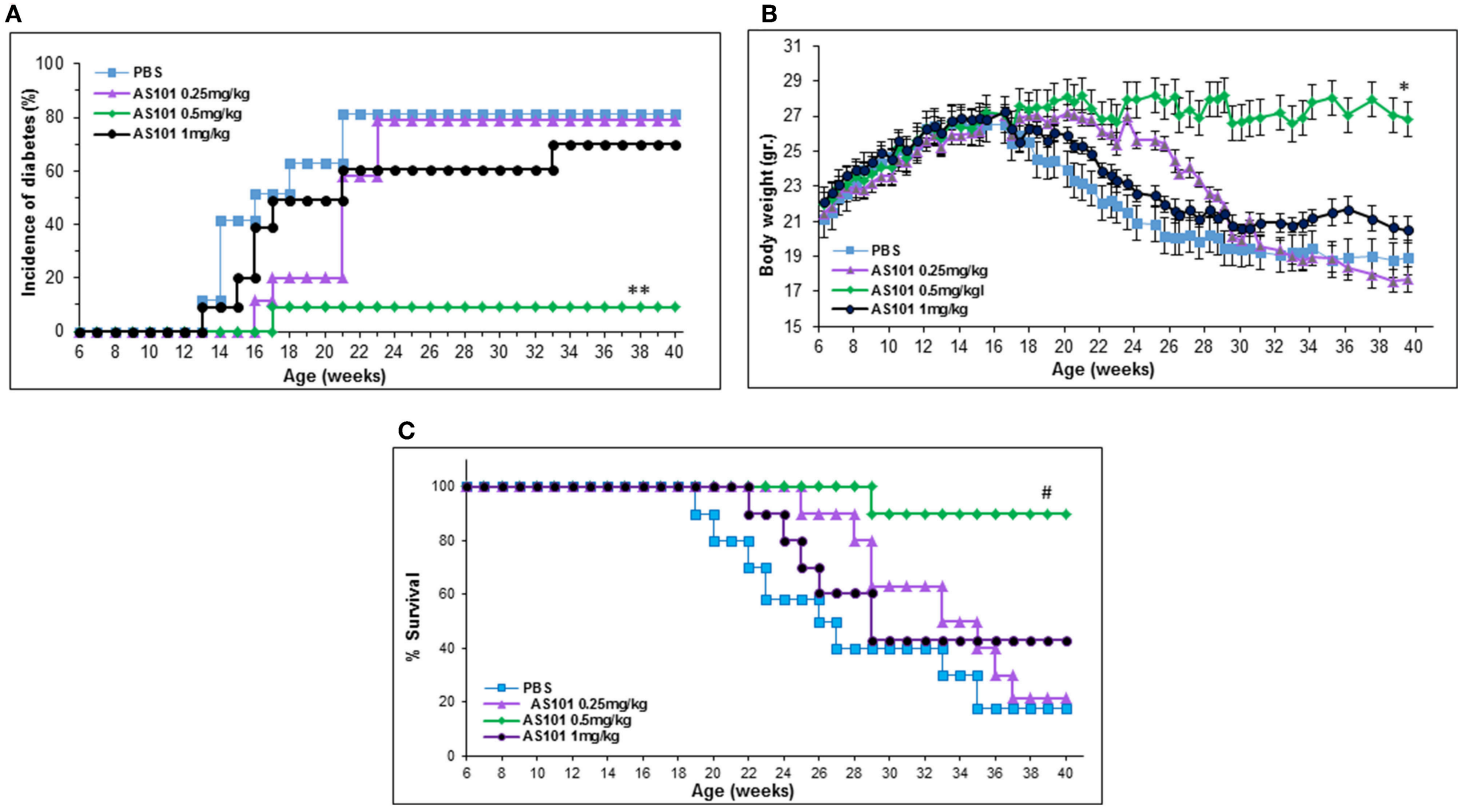
Treatment with AS101 starting at 5–6 weeks of age prevents the development of T1D in NOD mice. AS101 was administered i.p. every other day at different doses to female NOD mice, starting at 5–6 weeks of age, until 40 weeks. The incidence of diabetes (glucose levels at least 200 mg/dL **(A)**, body weight **(B)**, and percent survival **(C)** were monitored. *n* = 10 animals/group. ^**^p < 0.05 decrease vs. PBS; ^*^p < 0.05 increase vs. PBS; #*p* < 0.05 increase vs. PBS.

**Figure 6:**
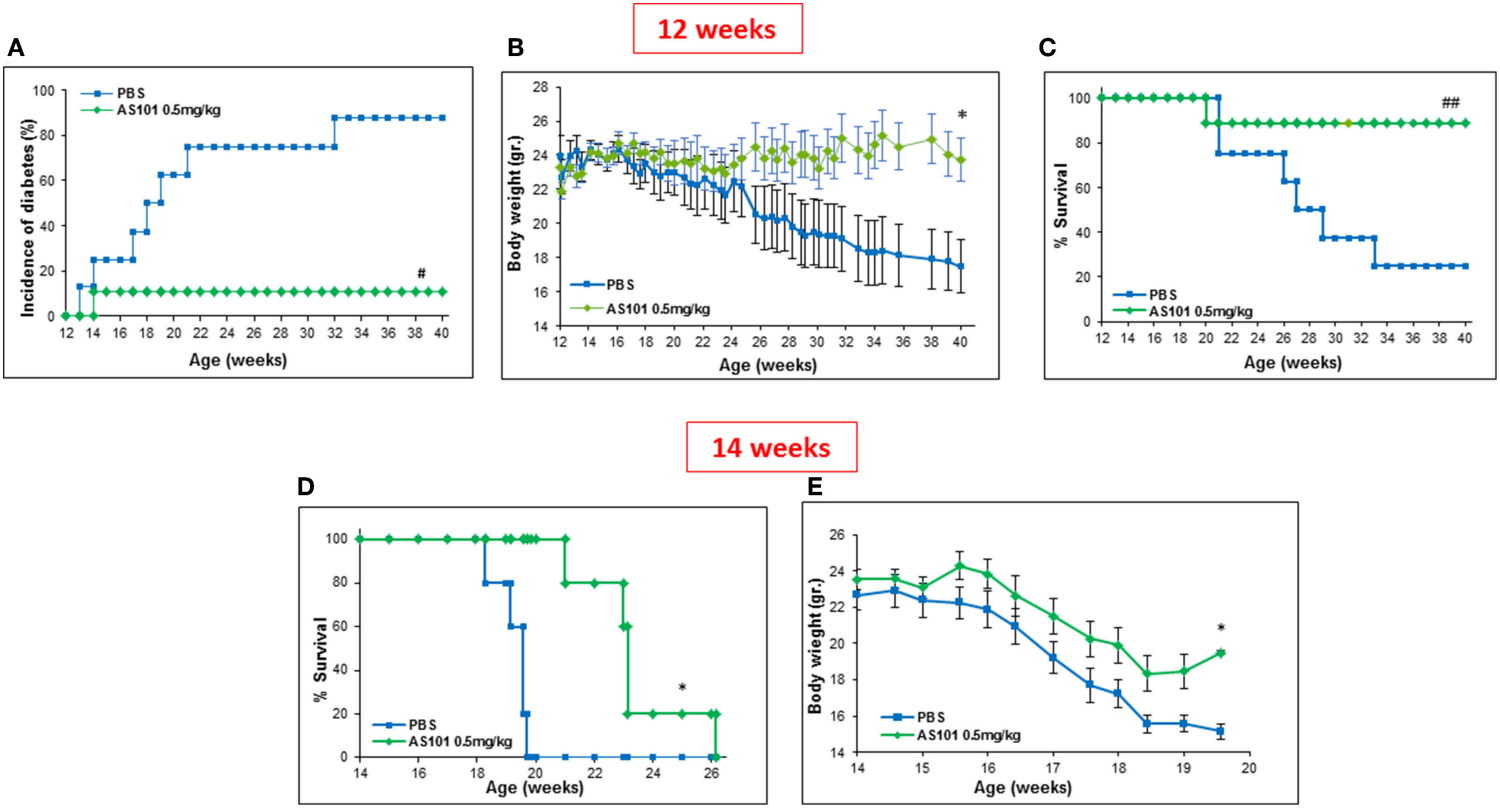
Treatment with AS101 starting at 12 weeks of age prevents the development of T1D in NOD mice. PBS or AS101 at 0.5 mg/kg/injection was administered i.p. every other day at different doses to female NOD mice, starting at 12 weeks of age, until 40 weeks. The incidence of diabetes **(A)**, body weight **(B)**, and percent survival **(C)** were monitored, *n* = 10/group. **(D,E)** AS101 at 0.5 mg/kg/injection was administered i.p. every other day at different doses to female NOD mice, starting at 14 weeks of age. Survival **(D)** and body weight **(E)** of mice was monitored. *n* = 5 mice/group. ##*p* < 0.005 increase vs. PBS. **#***p* < 0.01 decrease vs. PBS; ^*^p < 0.05 increase vs. PBS.

Figure 5A shows that extended treatment of NOD mice with an optimal dose of AS101 starting at 5–6 weeks (before the development of insulitis), resulted in the delay and prevention of diabetes incidence in a dose-dependent fashion, showing a narrow effective dose range (Figure 5). The optimal concentration of AS101 (0.5 mg/kg/injection) prevented disease in 90% of mice. Moreover, sub or supra-optimal concentrations of AS101 marginally delayed the onset of the disease, though these effects did not reach statistical significance This effect was accompanied by increased body weight (Figure 5B), and increased survival (Figure 5C) in mice receiving the optimal dose of 0.5 mg/kg. When treatment with AS101 at 0.5 mg/kg/mouse was stopped at 40 weeks of age, no change occurred with respect to percent incidence of diabetes, percent survival, or body weight until at least 52 weeks, suggesting a lasting and significant remission in the course of the disease. The average weight of mice at 52 weeks was 30 ± 77 gr, and 100% of mice surviving at 40 weeks, were also surviving with no diabetes at 52 weeks. None of the mice in the PBS control group survived to 52 weeks of age (data not shown). Importantly, even when AS101 was administered at an advanced stage of insulitis (12 weeks), the incidence of diabetes in treated mice until 40 weeks of age was only 10%, whereas that of control mice reached 90% at that time point (Figure 6A). This timing of AS101 administration resulted in preserved body weight (Figure 6B) and increased survival (Figure 6C). Importantly, even when treatment with AS101 at 0.5 mg/kg/mouse was started at 12 weeks and stopped at 40 weeks, all mice were remained alive, diabetes-free, and maintained their body weight at 52 weeks (average body weight of 26.88 ± 1.01 grams at 52 weeks). None of the mice in the control group were still alive at that time (data not shown). Moreover, not only could AS101 prevent development of diabetes when administered to pre-diabetic mice with different degrees of insulitis, but AS101 increased survival and body weight as well when administered to 14 w old mice that already developed symptoms of diabetes (all untreated NOD mice were diabetic at this time point, not shown) (Figures 6D,E).

### Treatment of NOD Mice With SAS Alleviates Clinical Symptoms of Diabetes

The encouraging results obtained regarding the beneficial effects of AS101 treatment on the course of T1D led us to investigate the potential effect of the new generation tellurium compound, SAS, shown here to inhibit the activity of α4β7 (Figures 2–4), on the intensity of disease. Treatment with SAS was compared to that with AS101 (the optimal dose of the specific batch of AS101 at that time was 1 mg/kg/injection).

**Figure 7:**
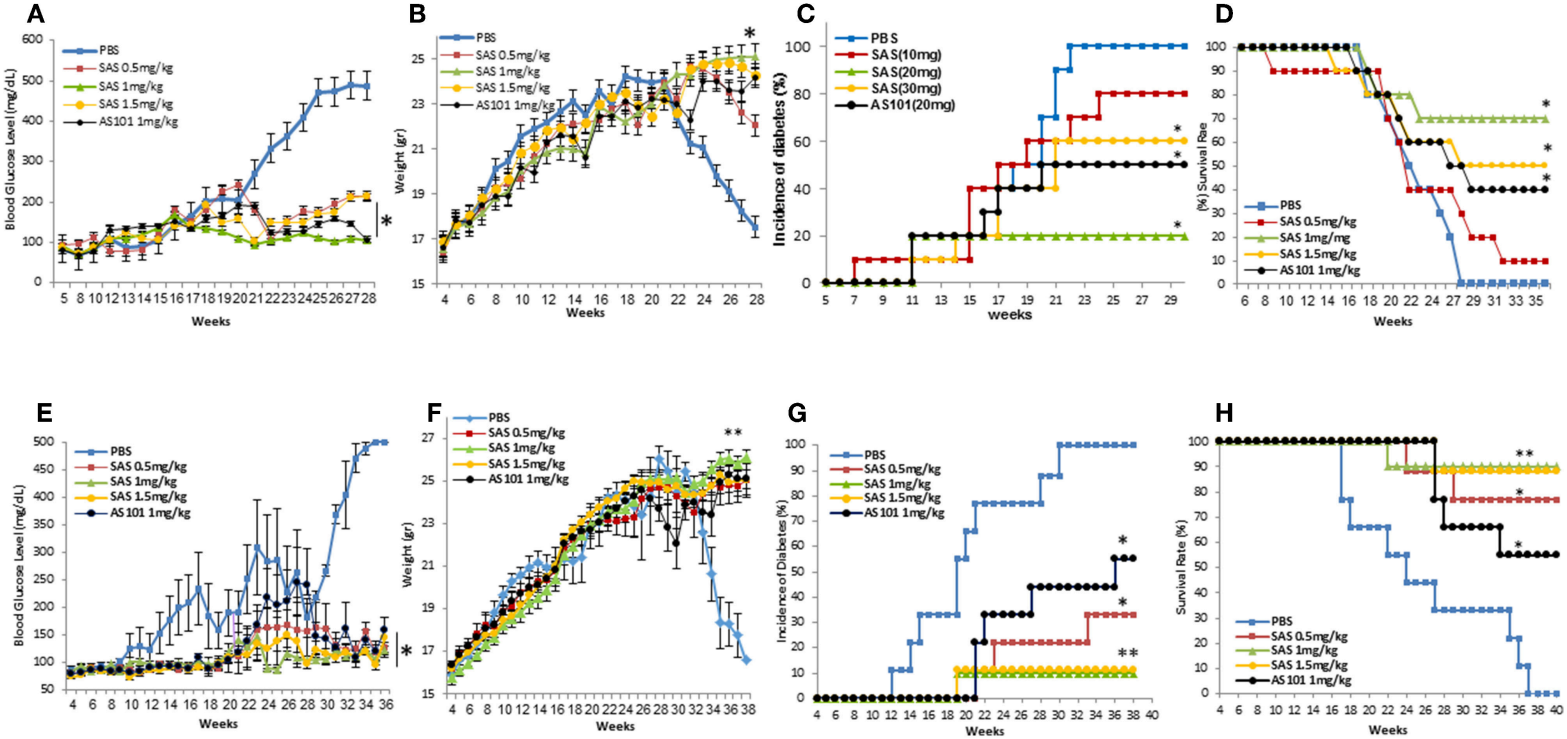
Treatment of NOD mice with SAS or AS101 decreases clinical symptoms of T1D in NOD mice. SAS at different concentrations or AS101 at 1 mg/kg/injection were administered i.p. every other day to female NOD mice, starting at either 5–6 weeks of age **(A–D)**, or at 3 weeks of age **(E-H)** until 30 weeks. Blood glucose levels (^*^p < 0.005 decrease vs PBS) **(A,E)**, body weight (^**^p < 0,001; ^*^p<*p* < 0.005 increase vs. PBS) **(B,F)**, percent incidence of diabetes (^*^p < 0.005 decrease vs. PBS; ^**^p < 0.001) **(C,G)**, and survival (^*^p < 0.005 increase vs. PBS) **(D,H)** were monitored. *N* = 10 mice/group.

Figure 7 shows that extended treatment with the tellurium compounds, AS101 and SAS of NOD mice starting at 5–6 weeks (Figures 7A–D) or at 3–4 weeks (Figures 7E–H) [initiation of the autoimmune process (23)], decreased levels of blood glucose (Figures 7A,E), decreased incidence of diabetes (Figures 7B,F), decreased weight loss (Figures 7C,G) and eventually increased survival (Figures 7D,H) (Figure 7). The results show that treatment with the tellurium compounds delay the onset of the disease, and even result in its prevention with increased efficiency when treatment is started earlier.

**Figure 8:**
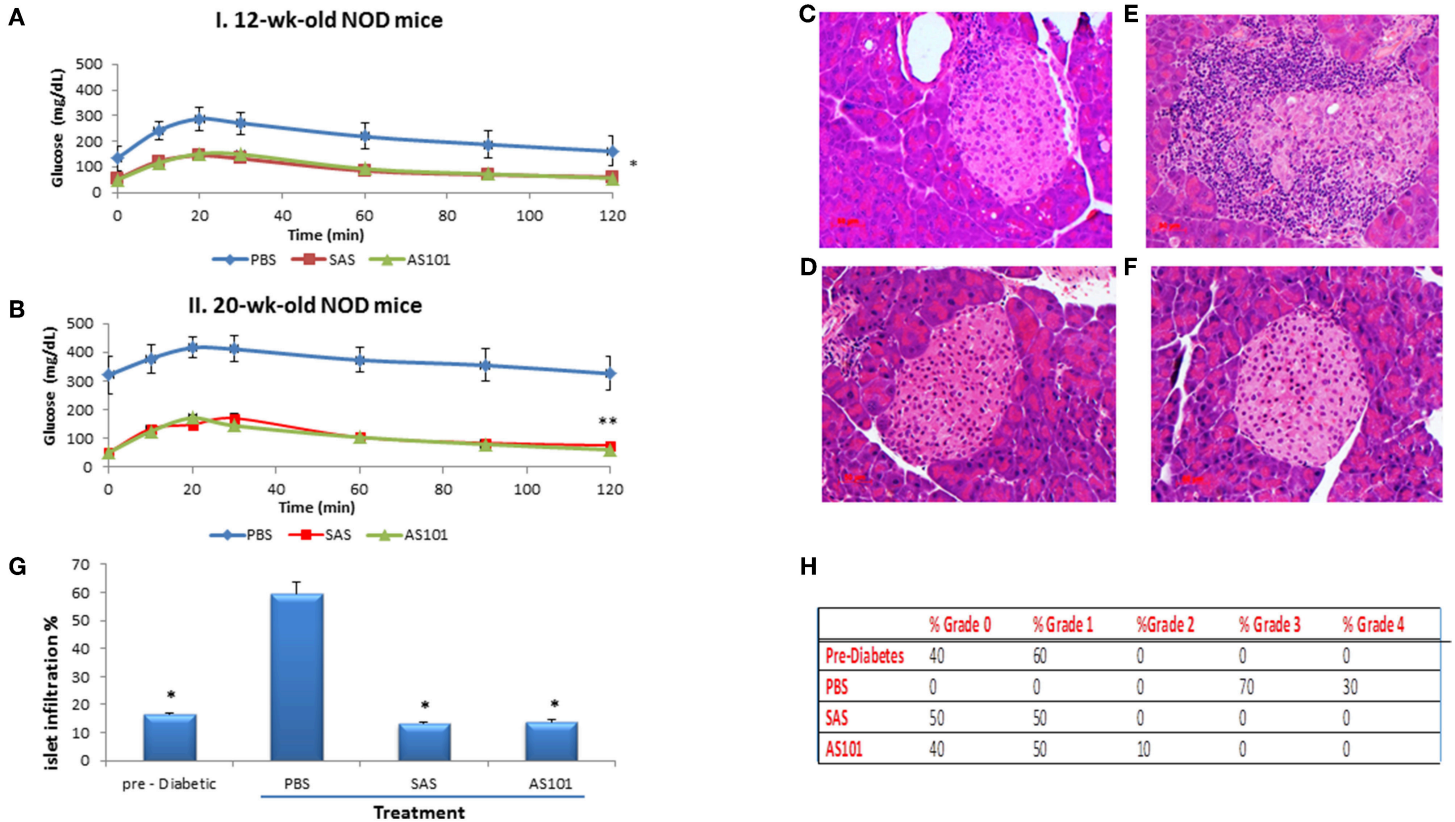
Effect of the compounds on islet morphology and insulitis. AS101 or SAS at 1mg/kg/injection were administered i.p. every other day to female NOD mice, starting at 3 weeks of age. At 12 weeks **(A)** (beginning of insulitis) or at 20 weeks **(B)** (severe diabetes)., mice were starved O.N. and injected i.p. with 2gr/kg glucose. Blood glucose levels were monitored at different time points. ^**^p < 0.01 vs. PBS; ^*^p < 0.05 vs. PBS. *N* = 10/group. **(C–F)** At 20 weeks, pancreatic heads were fixed and stained with H&E. **(C)** Pre-diabetic mice islets at 5w. **(D)** PBS. **(E)** AS101 (1 mg/kg/injection). **(F)** SAS (1 mg/kg/injection). Results show representative data of five mice/group. X200. **(G)** Percent insulitis of 5 mice/group at 20 weeks in comparison to islets from 5 weeks old mice (pre-diabetic). 20 islets/mouse were scored. ^*^p < 0.01 vs. PBS. **(H)** Percentage of infiltrating cells according to the severity of insulitis. Grade 0, No insulitis (no infiltration); Grade 1, Pre-insulitis (<25% of the islet area infiltrated); Grade 2, Mild insulitis (<50% of the islet area infiltrated); Grade 3, Severe insulitis (>50% of the islet area infiltrated); Grade 4, Massive insulitis (total loss of β cell mass).

**Figure 9:**
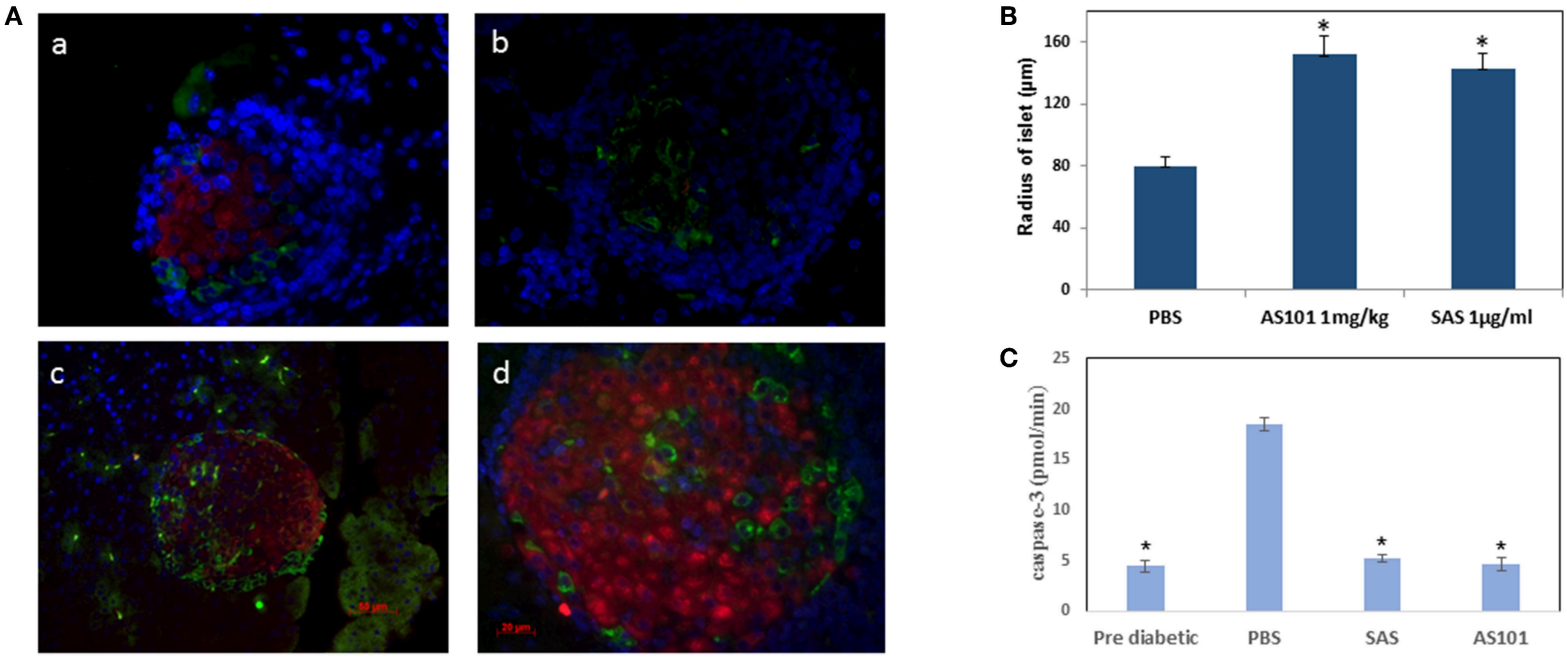
AS101 and SAS prevent islet damage and preserve islet function. **(A)**AS101 or SAS at 1 mg/kg/injection were administered i.p. every other day to female NOD mice, starting at 3 weeks of age. At 20 weeks, pancreatic heads were fixed and stained for insulin (red), glucagon (green), and nuclear staining (blue). Pancreata from 5 week old mice (pre-diabetic) served as a control. (a) Pre-diabetic; (b) PBS; (c) AS101; (d) SAS. The results are representative of 3 mice/group. 10 slides/mouse were visualized. X400. **(B)** Microscopic determination of islet radius. Results represent means ± SE of 5 mice/group. ^*^p < 0.01 vs PBS. **(C)** Pancreas cell lysates from 20w old mice were analyzed for caspase-3 activity by colorimetric assay. Results represent means ± SE of 5 mice/group. ^*^p < 0.01 vs. all groups.

Pretreatment with AS101 or SAS preserved optimal glucose tolerance (Figure 8). Treatment with AS101 and SAS resulted in normal levels of glucose in intraperitoneal glucose tolerance test (IPGTT), either when the test was performed at 12 w of age (Figure 8A), or at 20 w (Figure 8B), suggesting that preservation of pancreatic islets may have occurred in AS101 or SAS-treated mice early after treatment The fact that in treated mice, 1 h after glucose loading, glucose levels amounted to only ~100 mg/dL, considered to be normal levels, suggests that treatment with tellurium compounds prevents islet destruction, enabling normal function of β cells. Indeed, the results show that the level of insulitis was dramatically and significantly decreased in treated mice (Figures 8C–H) both with respect to the percentage of infiltrated islets (Figure 8G) and the percentage of cells with severe insulitis score (Figure 8H). Moreover, the function of the islets was preserved in treated mice, producing normal insulin in pancreatic islets (Figure 9A), as compared to mice in a pre-diabetic state. More importantly, in addition to the normal morphology and size of islets of treated mice (Figure 9B), caspase-3 activity, the classical hallmark of apoptosis, was significantly decreased compared to control mice, and not significantly different from that of pre-diabetic mice (Figure 9C).

### AS101 and SAS Attenuate Pancreatic Inflammation and Increase the Frequency of Tregs

**Figure 10:**
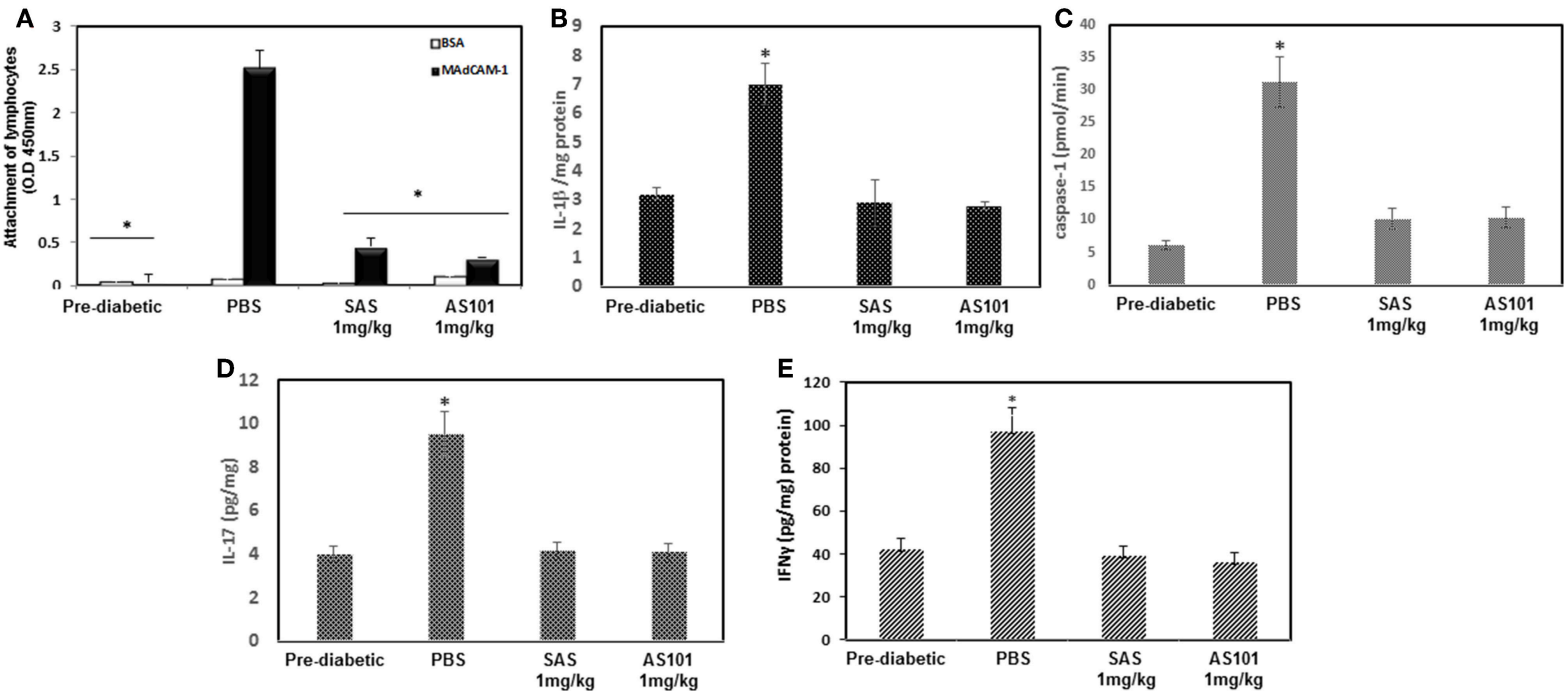
Treatment with AS101 or SAS attenuates α4β7 integrin activity and suppresses inflammatory cytokines in NOD mice. **(A)** AS101 or SAS at 1 mg/kg/injection were administered i.p. every other day to female NOD mice, starting at 3 weeks of age until 20 weeks. Pre-diabetic mice at 5 weeks of age served as controls. Lymphocytes from PAN LN at 5 × 10^5^/well were isolated and incubated in 96 well plates coated with BSA or MAdCAM-1 for 2 h. Nonattached cells were then washed twice, and the adherent cells were subjected to XTT. The results represent means ± SE of 8 mice/group. ^*^p < 0.01 vs. MadCAM-1 alone. **(B)** IL-1β protein levels from 20 w old mice. Pancreatic cell lysates were evaluated for IL-1β by ELISA. The results represent means ± SE of 5 mice/group. ^*^p < 0.01 vs. PBS. **(C)** At 20 weeks pancreas cell lysates were analyzed for caspase-1 activity by a colorimetric assay, IL-17 **(D)** and IFNγ **(E)** protein levels by ELISA. The results represent means ± SE of 3 mice/group. ^*^p < 0.001 vs. all.

In order to gain insight into the mechanism of the beneficial effects of both tellurium compounds in T1D, we first examined whether integrin α4β7 activity in PAN lymph nodes was inhibited following treatment. As can be seen in Figure 10A, the integrin activity in PLN lymphocytes from NOD mice treated with the compounds from 3 to 20 w was significantly inhibited. This was manifested by diminished attachment of the cells to the α4β7 specific ligand, MADCAM-1. This inactivation might prevent the infiltration of autoreactive cells to the pancreas.

**Figure 11:**
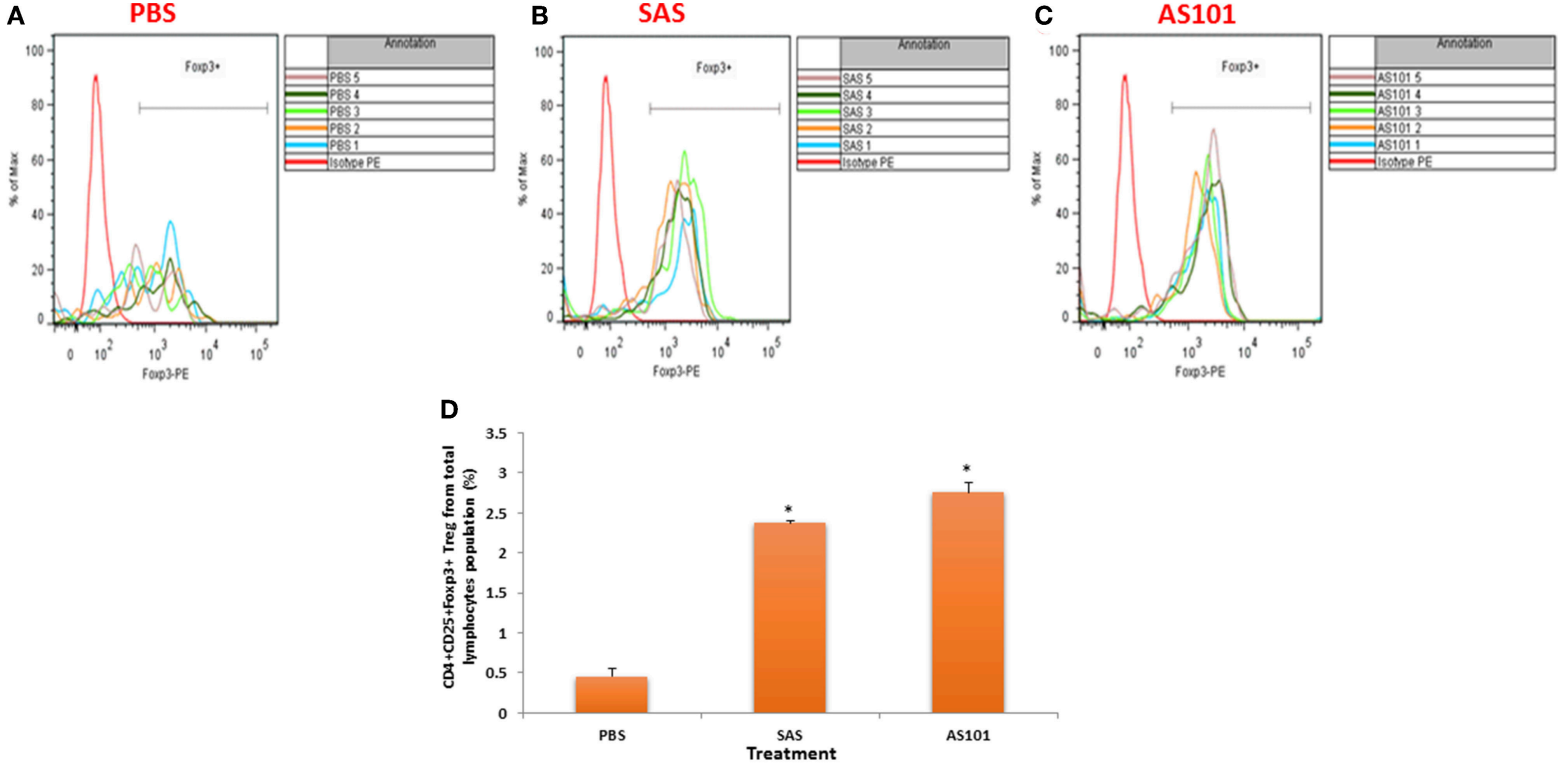
Treatment with AS101 or SAS increases the proportion of T regulatory cells in PLN of NOD mice. AS101 or SAS were administered i.p. at 1 mg/kg/injection every other day to female NOD mice, starting at 3 weeks of age. At 20 weeks lymphocytes from PLN were isolated and stained for CD4, CD25 and Foxp3. **(A)** PBS; **(B)** SAS (1 mg/kg/injection); **(C)** AS101 1mg/kg/injection. Each curve represents cells isolated from different animal *N* = 5/group. **(D)** Evaluation of the proportion of regulatory cells in treated mice. The results represent means ± SE of 5 mice/group.^*^p < 0.001 vs. PBS.

We next examined protein expression of IL-1β, a key proinflammatory cytokine, which drives several autoimmune diseases. IL-1 is a pleiotropic cytokine, which attenuates CD4^+^CD25^+^FoxP3^+^ regulatory T cell function, and allows the CD4^+^CD25^−^ autoreactive effector subset to escape from suppression (24). IL-1β has been previously shown to be inhibited by AS101 in various pre-clinical studies (14, 16, 25). Figure 10B shows that both AS101 and SAS significantly inhibited pancreatic IL-1 protein levels at 20 weeks. Furthermore, caspase-1 activity, previously shown to be inhibited by AS101 (16, 25) resulting in decreased IL-1β, was also inhibited in this study in PAN LN by AS101 and SAS-treated mice (Figure 10C). Importantly, the pro-inflammatory cytokine IL-17, which is implicated as essential in the development of T1D (26), was also inhibited in treated mice (Figure 10D). Furthermore, the Th1 cytokine IFNγ, playing a dominant role in the pathogenesis of autoimmune diabetes (27), was also significantly decreased in SAS and AS101-treated mice (Figure 10E). These results prompted us to evaluate the proportion of CD4^+^CD25^+^FoxP3^+^ regulatory T cells, known to be affected by these two inflammatory cytokines (IL-1β and IL-17). Figures 11A–D shows a significant increase in the proportion of Tregs among PAN LN cells.

**Figure 12:**
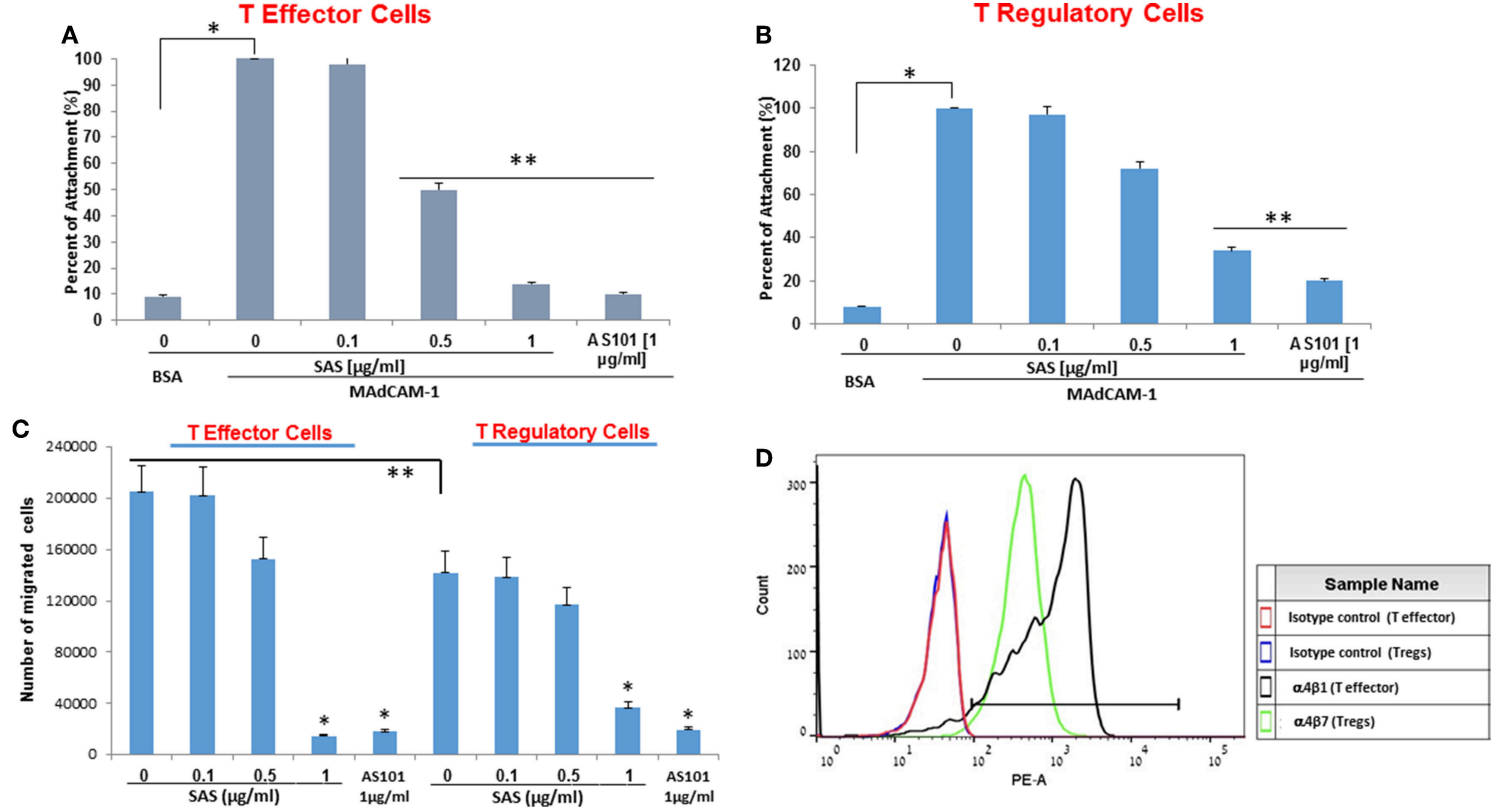
AS101 and SAS inhibit α4β7 integrin activity and α4β7-depenfent migration of T effector cells and Tregs. **(A)** T effector cells and **(B)** T regulatory cells were isolated from spleens of 20w old NOD female mice. Cells at 5 × 10^5^/well were incubated in 96 well plates coated with BSA or MAdCAM-1 for 2 hours. Nonattached cells were then washed twice, and the adherent cells were subjected to XTT. The results represent means ± SE of 4 mice/group. ^*^p < 0.001 vs. BSA; ^**^p < 0.005 vs. MAdCAM-1 alone (SAS 0), **(C)** T effector cells and T regulatory cells were isolated from spleens of 20w old NOD female mice. Cells (2.5 × 10^5^) were incubated for 1 h in the presence of mobilized MadCAM-1 and various doses of SAS or AS101 and then loaded onto 8 mm polycarbonate membrane inserts. The bottom chambers were filled with FBS (20%) and MadCAM-1 (100 μg/ml) serving as a chemo attractant. Migrated cells were quantified after 24 h. The results represent means±SE of 4 mice/group. ^*^p < 0.001 vs control (SAS 0 of the relevant cell subset). ^**^p < 0.05 (comparison between controls of both cell subsets). **(D)** Tregs and T effector cells were isolated from spleens of 20w old female NOD mice and stained for the expression of α4β7. The results represent one experiment representative of three performed with 3 mice/group.

The increase in Tregs following treatment by SAS or AS101 prompted us to evaluate if the tellurium compounds affect the α4β7 integrin activity in Tregs vs. T effector cells and to explore the effects and if this property affects the migration of each subset of cells. Figures 12A,B shows that SAS and AS101 inhibits the activity of the α4β7 integrin in both isolated T effector cells (Figure 12A) and regulatory cells (Figure 12B); nevertheless, the extent of inhibition is higher in T effector cells. Moreover, the α4β7 integrin-dependent migration of both T cell subsets was also inhibited by both compounds, however that of T effector cells was more intense. Furthermore, The extent of control T regulatory cells migration was significantly lower than that of T effector cells (Figure 12C). Interestingly, the expression of a4b7 in T regulstoty cells was lower than that of T effector cells (Figure 12D). This might explain the lower a4b7-dependent of Tregs and their decreased sensitivity to tellurium compound inhibition.

Finally, we wished to evaluate if offspring of AS101-treated NOD mothers acquire some resistance to T1D development. Female NOD mice were treated with AS101 starting 2 weeks prior to mating, during pregnancy and throughout breast feeding. When the pups were analyzed, they exhibited decreased incidence of diabetes (Supplementary Figure 1A) and increased survival (Supplementary Figure 1B). Moreover, offspring of treated mothers were relatively more resistant to adoptive transfer of autoreactive cells as expressed by decreased incidence of diabetes (Supplementary Figure 1C) and increased survival (Supplementary Figure 1D). These results suggest that AS101 can significantly protect the fetus from future diabetes development when the mother is treated during pregnancy. Furthermore, the fetus might acquire both diminished autoreactive cells and increased regulatory mechanisms.

## Discussion

The present study presents evidence demonstrating the beneficial effects of the two non-toxic tellurium small molecules AS101 and SAS in a mouse model of T1D. Systemic treatment with these compounds could preserve β cells function and mass. These beneficial effects were reflected in decreased incidence of diabetes, decreased hyperglycemia, improved glucose clearance, preservation of body weight and increased survival. The normal glucose levels were associated with increased insulin levels, preservation of β cell mass and increased islet size compared to controls. Importantly, this protective activity could be demonstrated when the compounds were administered either at the early pre-diabetic phase with either no or early insulitis, at the pre-diabetic stage with advanced insulitis, or even at the advanced, overtly diabetic stage. Moreover, delaying the onset of diabetes or its prevention was dose-dependent, showing an optimum curve.

Our results suggest that both tellurium compounds prevent migration of autoimmune lymphocytes to the pancreas, i.e., prevent insulitis, by inhibiting α4β7 integrin activity. Indeed, the decreased infiltration resulted in a significantly diminished extent of pancreatic islet damage, both with respect to their size, β cell function (insulin production), and caspase-3 activity, the hallmark of apoptosis. Most importantly AS101 and SAS significantly elevated the frequency of T regulatory cells in the pancreas, thus potentially controlling the autoimmune process.

We have shown in our previous studies that much of the biological activity of AS101 and SAS is directly mediated by their unique chemical interactions with specific cysteine thiol residues. The formation of such TeIV-thiol bonds may induce conformational changes or alter disulfide bond formation in specific proteins, possibly resulting in the loss of their biologic activity, in cases where the thiol residue is essential for a particular function (28, 29). Much of the biological activity of AS101 may be directly attributed to its redox interactions with vicinal thiols in the exofacial of the α4β1 integrinVLA-4 (10), residing within the α but not the β chain of the integrin. We thus suggested that the α4β7 integrin sharing the same α4 chain might be similarly inhibited by the tellurium compounds. This also implies that the specific thiol residues on the α4 chain of the α4β7 integrin are essential for the α4β7 integrin activity. Indeed, we previously showed that AS101 inhibits the α4β7 activity in mesenteric lymph node cells of DSS-induced murine colitis (14). This study shows for the first time that both tellurium compounds suppress the activity of the α4β7 integrin in pancreatic LNs, impacting lymphocyte infiltration into PanLN, and consequently affecting the pathogenesis of T1D.

It was previously shown that the mucosal vascular addressin MAdCAM-1, the ligand for α4β7, is also constitutively expressed at low levels on pancreatic vasculature and, in conjunction with the appearance of lymphocyte infiltrates (insulitis) in pancreatic islets, becomes strongly induced on islet vessels (30–32). Indeed, we show in the present work that both tellurium compounds inhibit the attachment of lymphocytes from pancreatic lymph nodes of NOD mice to either rMAdCAM-1 or to MAdCAM-1 expressed on endothelial cells after exposure to TNFα. This inhibition was demonstrated both *in vitro* and *in vivo*. MAdCAM - 1 is expressed on the surface of endothelial cells in response to secretion of inflammatory cytokines such as to secretion of inflammatory cytokines, including Tumor necrosis alpha (TNF-α) and Interleukin-1 beta (IL-1β). We found that mice successfully treated with AS101 or SAS, showed a significant decrease in pancreatic IL-1β, a cytokine that plays an important role in many autoimmune diseases (33). The decrease in Il-1β suggests that AS101 and SAS prevent insulitis not only by inhibiting the activity of the α4β7 integrin but also possibly by indirectly inhibiting the expression MAdCAM-1 on pancreatic vasculature via inhibition of IL-1β. This cytokine has been previously shown to be inhibited by the tellurium compounds in various preclinical studies due to inhibition of caspase-1 activity (14, 16, 26). Interestingly, we have recently shown that modulation of caspase-1 activity is dependent on VLA-4 inactivation and is mediated through VLA-4 inactivation, and underlies the anti-inflammatory activity of AS101 (16). In line with this, the present study shows a significant decrease in pancreatic caspase-1 activity. The inhibition of α4β7 activity by both Tellurium compounds was most evident both in T effector cells an Treg cells, although that of Tregs was lower. This might be explained by the diminished expression of the a4b7 integrin in Tregs.

It has been previously reported that IL-1β induces proliferation and cytokine production by CD4^+^CD25^+^FoxP3^−^ effector/memory T cells, attenuates CD4^+^CD25^+^FoxP3^+^ regulatory T cell function, and releases CD4^+^CD25^−^ autoreactive effectors from physiological suppression (24). Thus, inflammation or constitutive overexpression of IL-1β in a genetically predisposed host can promote activation and expansion of autoreactive effector T cells, thereby interfering with the ability of Treg cells to maintain self-tolerance. Thus, inhibition of of IL-1β by AS101 and SAS might explain the upregulation of Tregs by the compounds.

Multiple studies support an essential role of Th17 cells together with defects in T regulatory (Treg) cell function in the development of T1D in both animal models and in humans (34). The decrease in pancreatic Th17 in AS101 or SAS treated mice can be explained in two ways. IL-1β is known to promote IL-17 production by memory CD4^+^ T cells (35). Hence, its inhibition by the tellurium compounds might affect IL-17 levels. Conversely, the increase in regulatory cells could regulate Th17 cells. The potential pathways through which Treg cells regulate the Th17 cell response have been elucidated in several studies. In mice, CD4^+^ Treg cells restrain the Th17 immune response via Foxp3 binding to STAT3, a key factor in the initiation of Th17 differentiation (36). Overexpression of Foxp3 results in a strong reduction of IL17A gene expression by inhibiting RORγt-mediated IL-17A mRNA transcription (37). On the other hand, Th17 cells counteract the Treg cells, to expand and allow the development of T1D (26). Thus, the homeostasis between Th17 and Tregs is important in keeping autoimmunity in check. Our results showing a delay of the onset of diabetes by AS101 and SAS combined with their effect on IL-17 and Tregs imply they have the ability to preserve this homeostasis and regulate autoimmunity. Interestingly, although the tellurium compounds inhibited α4β7-dependent migration of both effector cells and Tregs, the proportion of Tregs in PLNs was increased in treated mice. We assume that this this may be explained by our results showing that the extent of inhibition is lower in Tregs as opposed to T effector cells, possibly because of the lower expression of α4β7 on Tregs (Figure 12D). Furthermore, even if less Tregs arrived to pancreatic lymph nodes, their number would be much increased with time within their low IL-1β and IL-17 environment.

Several clinically relevant observations emerged from this study:

1. When treatment with AS101 was stopped at 40 weeks of age, no change occurred with respect to incidence of diabetes, survival, or body weight at least until 52 weeks, suggesting that this compound had a long lasting effect on preventing T1D.
2. AS101 could alleviate symptoms of the disease even when treatment was started at relatively advanced stages of the disease (12 or 14 weeks of age). While T1D can be predicted in human patients based on the presence of antibodies recognizing autoantigens, such as insulin, GAD65 (glutamic acid decarboxylase), IA-2 (islet antigen 2), and ZnT8 (islet zinc transporter) (38), there is no known way to prevent or arrest the development of this disease, nor has any treatment been demonstrated that can reduce the anti-β-cell autoimmune response following the diagnosis of diabetes. Therefore, our positive results in the early treatment protocol (starting at 3 weeks of age) are applicable to individuals at risk, in which the disease is predicted by the presence of autoantibodies. Conversely, at more advanced stages of disease, AS101 might enable regeneration of β cells or their preservation. Indeed, it has been previously suggested that β cell regeneration can be induced in animal models provided a sufficient endogenous β cell mass remains (39, 40).
3. The increased resistance to diabetes followed by the increased survival could be transferred to offspring of 5w old female NOD mice treated with AS101 starting 2 weeks prior to gestation, during pregnancy and throughout breast feeding (Supplementary Figure 1). These results suggest that AS101 can significantly protect the fetus from future diabetes development when the mother is treated during pregnancy. The fact that decreased incidence of diabetes occurs in offspring of treated mothers undergoing adoptive transfer of autoreactive cells suggests that these offspring acquire from the mother regulatory means to fight the autoimmune cells injected. Thus, not only the fetus but the newborn as well can benefit from treatment of his mother. It has been recently reported that breastfeeding transfers to the infant not only protective antibodies but also maternal immune cells (41). The mechanisms underlying these important results are now the focus of our laboratory studies.

In summary, we introduce in the present study two non-toxic small molecules that, by virtue of their unique mechanism of action, may both prevent trafficking of autoreactive B and T cells to the pancreas and regulate the activity of those that have infiltrated the tissue. As AS101 demonstrated an excellent safety profile in clinical trials for other indications, these compounds may serve as good candidates for the treatment of T1D.

## Ethics Statement

Experiments conformed to approved institutional protocols and were approved by the Institutional Animal Care and Use Committee.

## Author Contributions

TY and ZB performed and analyzed the experiments. DK-S provided technical assistance. YK, RC, and BS designed the study, discussed the results, and wrote the paper. All the authors discussed the results and commented on the manuscript.

### Conflict of Interest Statement

The authors declare that the research was conducted in the absence of any commercial or financial relationships that could be construed as a potential conflict of interest.

## Supplementary Material

Supplementary Figure 1 The decreased incidence of diabetes followed by the increased survival can be transferred to offspring of treated female NOD mice. 5 w old female mice were treated with PBS or with various concentrations of AS101. Treatment continued during gestation and pregnancy and during breast feeding until the offspring aged 3 weeks. At that time treatment stopped and female offspring were evaluated for % incidence of diabetes **(A)** and for % survival **(B)** from 10 to 40 weeks. *N* = 15 offspring mice/group. ^*^*p* < 0.05; ^**^*p* < 0.005 vs. PBS. Some of the male offspring of treated mothers were irradiated at 6w of age with 550Gy and were injected 24h later with 20 × 10^6^ splenocytes from diabetic female NOD mice. The incidence of diabetes **(C)** and the percentage of survival **(D)** were monitored until 30 weeks.^*^*p* < 0.05 vs. PBS. N-9.
