# Supplementary figures and images for: Tellurium Compounds Prevent and Reverse Type-1 Diabetes in NOD Mice by Modulating α4β7 Integrin Activity, IL-1β, and T Regulatory Cells
Source: Front Immunol. 2019 May 29;10:979. doi: 10.3389/fimmu.2019.00979 (PMC6549385; doi:10.3389/fimmu.2019.00979)

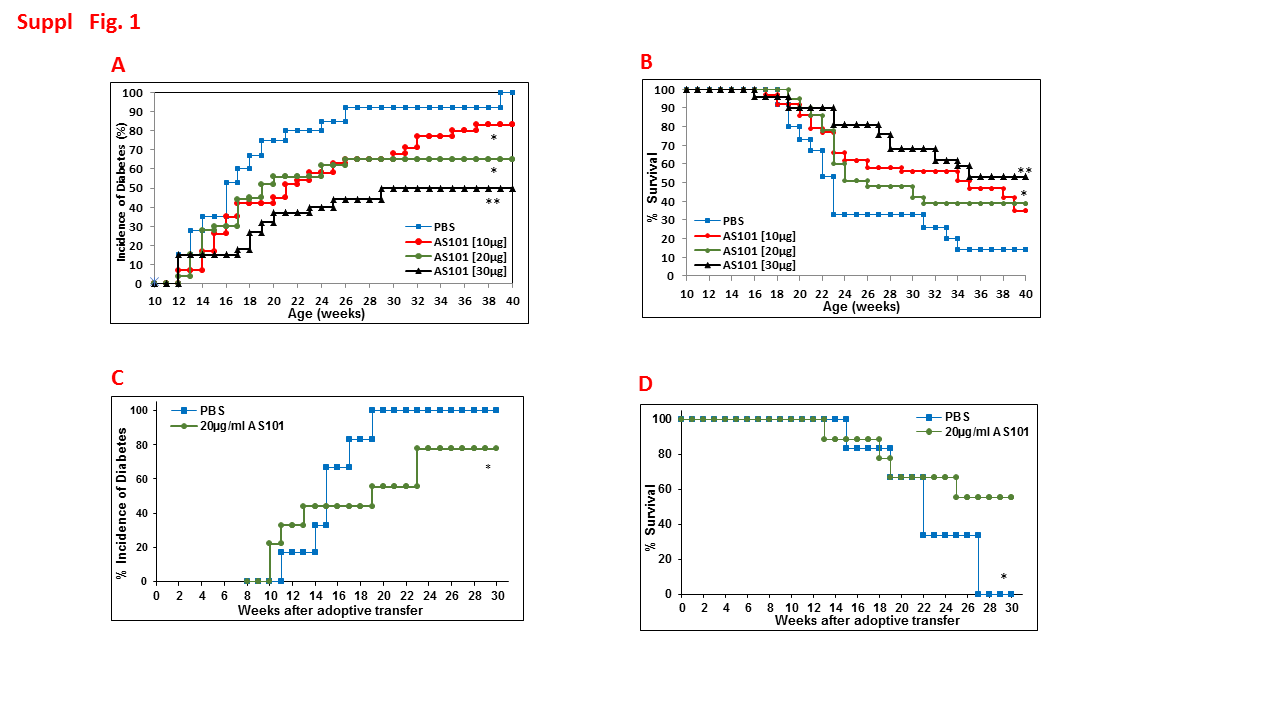

Supplement: Supplementary Figure 1 — The decreased incidence of diabetes followed by the increased survival can be transferred to offspring of treated female NOD mice. 5 w old female mice were treated with PBS or with various concentrations of AS101. Treatment continued during gestation and pregnancy and during breast feeding until the offspring aged 3 weeks. At that time treatment stopped and female offspring were evaluated for % incidence of diabetes (A) and for % survival (B) from 10 to 40 weeks. N = 15 offspring mice/group. *p < 0.05; **p < 0.005 vs. PBS. Some of the male offspring of treated mothers were irradiated at 6w of age with 550Gy and were injected 24h later with 20 × 106 splenocytes from diabetic female NOD mice. The incidence of diabetes (C) and the percentage of survival (D) were monitored until 30 weeks.*p < 0.05 vs. PBS. N-9. [file Image_1.TIF]
